# The prognostic value of long non coding RNAs in cervical cancer: A meta-analysis

**DOI:** 10.18632/oncotarget.17620

**Published:** 2017-05-04

**Authors:** Xiangrong Cui, Xuan Jing, Xueqing Wu

**Affiliations:** ^1^ Reproductive Medicine Center, Children's Hospital of Shanxi and Women Health Center of Shanxi, Affiliate of Shanxi Medical University, Taiyuan, 030000, P.R. China; ^2^ Clinical Laboratory, Shanxi Provincial People's Hospital, Affiliate of Shanxi Medical University, Taiyuan, 030012, P.R. China

**Keywords:** lncRNA, cervical cancer, prognosis, biomarker, meta-analysis

## Abstract

Cervical cancer is one of the most common malignancies in women worldwide. Numerous literatures demonstrate that aberrantly expressed lncRNAs are involved in tumorigenesis and development, and may have the potential to be prognostic markers. However, their prognostic functions in cervical remain controversial. Therefore, we performed this meta-analysis to evaluate the prognostic significance of lncRNAs in cervical cancer. We searched databases to identify relevant articles. Pooled hazard ratios (HRs) and 95% confidence intervals (CIs) were calculated. 15 studies involving 1868 patients with cervical cancer and 12 lncRNAs were included. Our results indicated that the levels of lncRNAs were associated with the overall survival ((HR = 1.56, 95%CI = 1.02–2.38, *P* < 0.001, random-effect) and event free survival (HR = 1.33, 95%CI = 0.77–2.28, *P* < 0.01, random-effect). High HOTAIR expression was associated with shorter overall survival in cervical cancer (HR = 3.93, 95% CI = 2.34–6.62, *P* < 0.001, fixed-effect). In conclusion, our meta-analysis suggested that lncRNAs may serve as novel predictive factors for prognosis of cervical and high expression HOTAIR was associated with shorter overall survival in cervical cancer.

## INTRODUCTION

Cervical cancer is the second most deadly gynecological cancer and caused about 260,000 women’s death each year [1]. Radical surgery is recommended as the preferred treatment for patients with cervical cancer in early stage, producing a relatively favorable prognosis [2]. However, patients with cervical cancer are usually diagnosed at advanced or recurrent stages with relatively poor prognosis [3]. Hence, exploring sensitive and specific biomarkers for prognosis is critical for the research and clinical treatment of cervical cancer.

Long noncoding RNA (lncRNA) are a kind of non-coding transcripts longer than 200 nucleotides [4]. LncRNAs can regulate various biological processes, such as gene expression, transcription, and cellular proliferation, through epigenetic silencing, mRNA splicing, and lncRNA-microRNA interaction [5, 6]. Furthermore, by comparing their expression of tumors and normal cells, lncRNAs are abnormally expressed in the various tumors, functioning as oncogenes or tumor suppressors [7, 8]. Recent studies have shown that lncRNAs also play important role in cervical cancer, including occurrence, progression, metastasis and prognosis [9, 10]. However, due to the sample size and research programs, singe study may be inaccurate and insufficient. With the aim to obtain a better understanding of the prognostic value of lncRNAs in patients with cervical cancer, we performed a meta-analysis to explore the prognostic value of lncRNAs through larger sample size of patients.

## RESULTS

### Study characteristics

**Figure 1:**
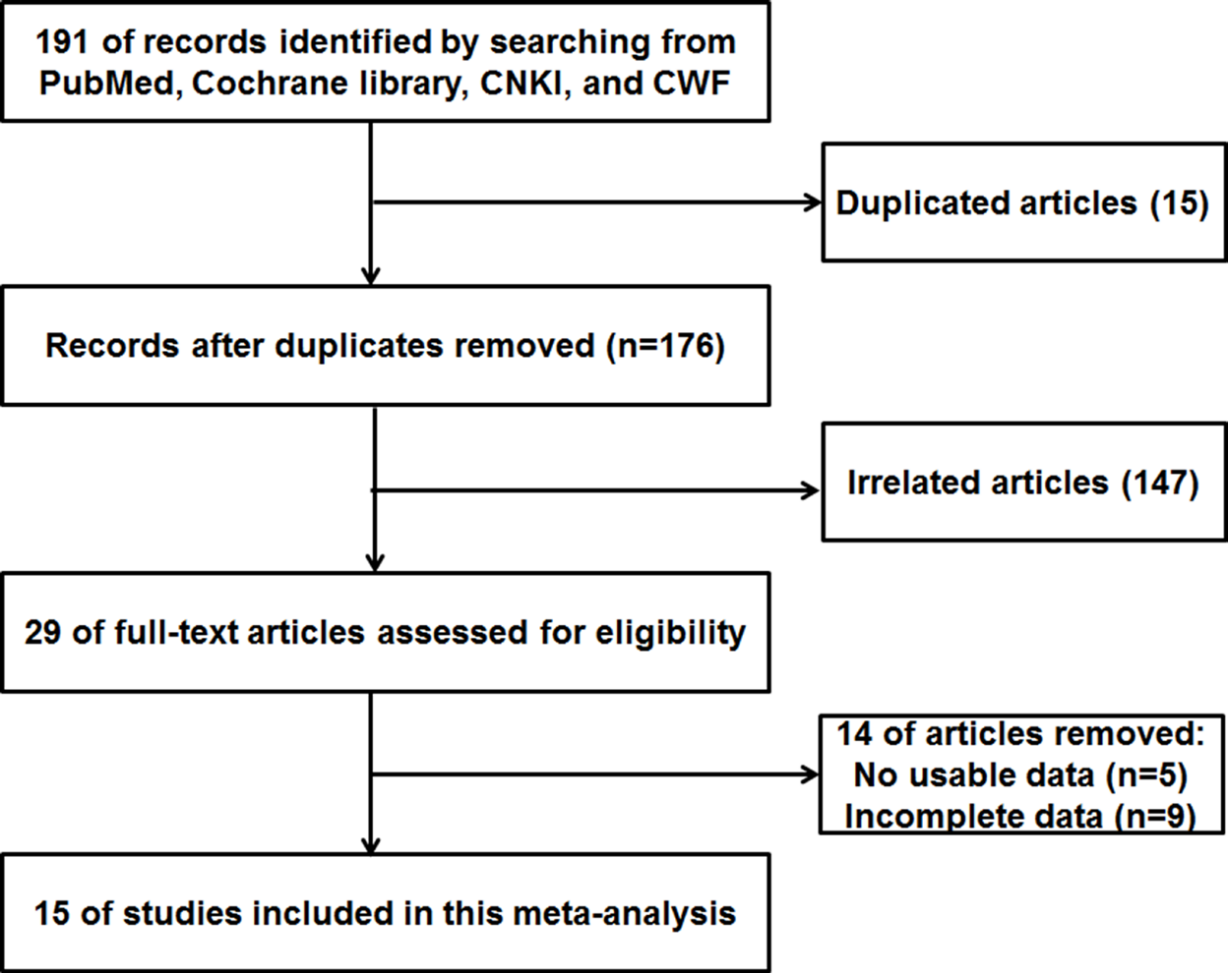
The flow diagram indicated the process of study selection

As shown in Figure 1, we searched 191 articles in the databases. After screening the titles and abstracts, 29 full-text articles were assessed for eligibility. Then because of no usable data or incomplete data, 14 papers were excluded. As a result, a total of 15 articles were the current meta-analysis [11–25].

The basic information and data from the included studies are shown in Table 1 and Table 2. 15 studies enrolling 1868 participants, with a maximum sample size of 218 and a minimum sample size of 49 patients. Among these 15 studies, 14 focused on Asians and 1 evaluated Caucasians. 9 studies reported patient overall survival (OS), 2 focused on OS as well as disease free survival (DFS), and 5 investigated OS as well as relapse free survival (RFS). DFS and RFS were combined together into event free survival (EFS) as prognosis parameter of our study. All studies investigated patients with cervical cancer and qRT-PCR was performed to detect lncRNAs expression in tumor tissues. Because the cut-off definitions were various, the cut-off values were different in these studies.

**Table 1:** Basic information of included studies

Study ID	LncRNA	Country	Dominant ethnicity	Sample	Reference	Detection method	Sample size	Outcome	Source of HR	Cut off value
Lingmin Liao 2014	XLOC_010588	China	Asian	tissue	GAPDH	qRT-PCR	218	OS/DFS	Reported&SC	median
Long Huang 2014	HOTAIR	China	Asian	tissue	GAPDH	qRT-PCR	218	OS/DFS	Reported&SC	median
Jing Li 2014	HOTAIR	China	Asian	tissue	GAPDH	qRT-PCR	118	OS/RFS	SC	median
Shihong Cao 2014	GAS5	China	Asian	tissue	RNU6B	qRT-PCR	102	OS	Reported	0.29
L. Yang 2015	MALAT1	China	Asian	tissue	GAPDH	qRT-PCR	104	OS/RFS	Reported&SC	3.075
Meng Yang 2015	CCHE1	China	Asian	tissue	RPS18	qRT-PCR	182	OS/RFS	SC	median
Reiko Kobayashi 2015	XIST	Japan	Asian	tissue	GAPDH	qRT-PCR	49	OS	Reported	median
Hee Jung Kim 2015	HOTAIR	Korea	Asian	tissue	U6	qRT-PCR	111	OS	Reported	30-fold
Shan Jiang 2015	LET	China	Asian	tissue	GAPDH	qRT-PCR	94	OS	Reported	mean
Y.F. Wang 2016	HULC	China	Asian	tissue	GAPDH	qRT-PCR	244	OS	Reported	median
Shaorong Zhang 2016	PVT1	China	Asian	tissue	GAPDH	qRT-PCR	90	OS	SC	median
Marissa Iden 2016	PVT1	America	Caucasian	tissue	RPS18	qRT-PCR	121	OS	SC	median
Hee Jung Kim 2016	HOXA11	Korea	Asian	tissue	U6	qRT-PCR	92	OS	Reported	227.5-fold
Jun Zhang 2017	MEG3	China	Asian	tissue	β-actin	qRT-PCR	72	OS/RFS	Reported&SC	median
Dongli Zhang 2017	ANRIL	China	Asian	tissue	GAPDH	qRT-PCR	53	OS	Reported	median

**Table 2:** Summary of hazard ratios of lncRNA expression in cervical cancer

lncRNAs	Reference	Case number			OS		DFS/RFS	
		High expression	Low expression	HR (95% CI)		*P* Value	HR (95% CI)	*P* Value
HOTAIR	Long Huang, 2014	109	109	4.57 (2.12–9.85)		< 0.001	2.98 (1.62–5.45)*	0.0004
HOTAIR	Jing Li, 2014	59	59	3.15 (1.44–6.88)*		0.004	2.11 (1.18–3.77)*	0.012
HOTAIR	Hee Jung Kim, 2015	89	22	5.28 (1.01–27.75)		0.049	NM	NM
PVT1	Shaorong Zhang, 2016	45	45	1.97 (1.14–3.40)*		0.015	NM	NM
PVT1	Marissa Iden, 2016	63	58	1.73 (1.05–2.84)*		0.03	NM	NM
XLOC_010588	Lingmin Liao, 2014	109	109	0.37 (0.17–0.75)		0.006	0.45(0.25–0.83)*	0.008
GAS5	Shihong Cao, 2014	58	44	3.22 (1.68–6.17)		< 0.001	NM	NM
MALAT1	L. Yang, 2015	52	52	2.21 (1.08–4.56)		0.031	2.60 (1.60–4.23)*	0.0001
CCHE1	Meng Yang, 2015	91	91	2.27 (1.30–3.96)*		0.004	1.23 (1.10–1.38)*	0.0004
XIST	Reiko Kobayashi, 2015	24	25	0.27 (0.08–0.86)		0.027	NM	NM
LET	Shan Jiang, 2015	44	50	0.40 (0.22–0.73)		0.003	NM	NM
HULC	Y.F. Wang, 2016	120	124	1.88 (1.29–2.74)*		0.001	NM	NM
HOXA11	Hee Jung Kim, 2016	41	51	2.45 (1.08–5.56)		0.032	NM	NM
MEG3	Jun Zhang, 2017	36	36	0.33 (0.15–0.72)*		0.005	0.16 (0.03–0.92)	0.04
ANRIL	Dongli Zhang, 2017	27	26	3.23 (1.42–7.32)*		0.005	NM	NM

### Analysis between lncRNAs expression level and OS

**Figure 2:**
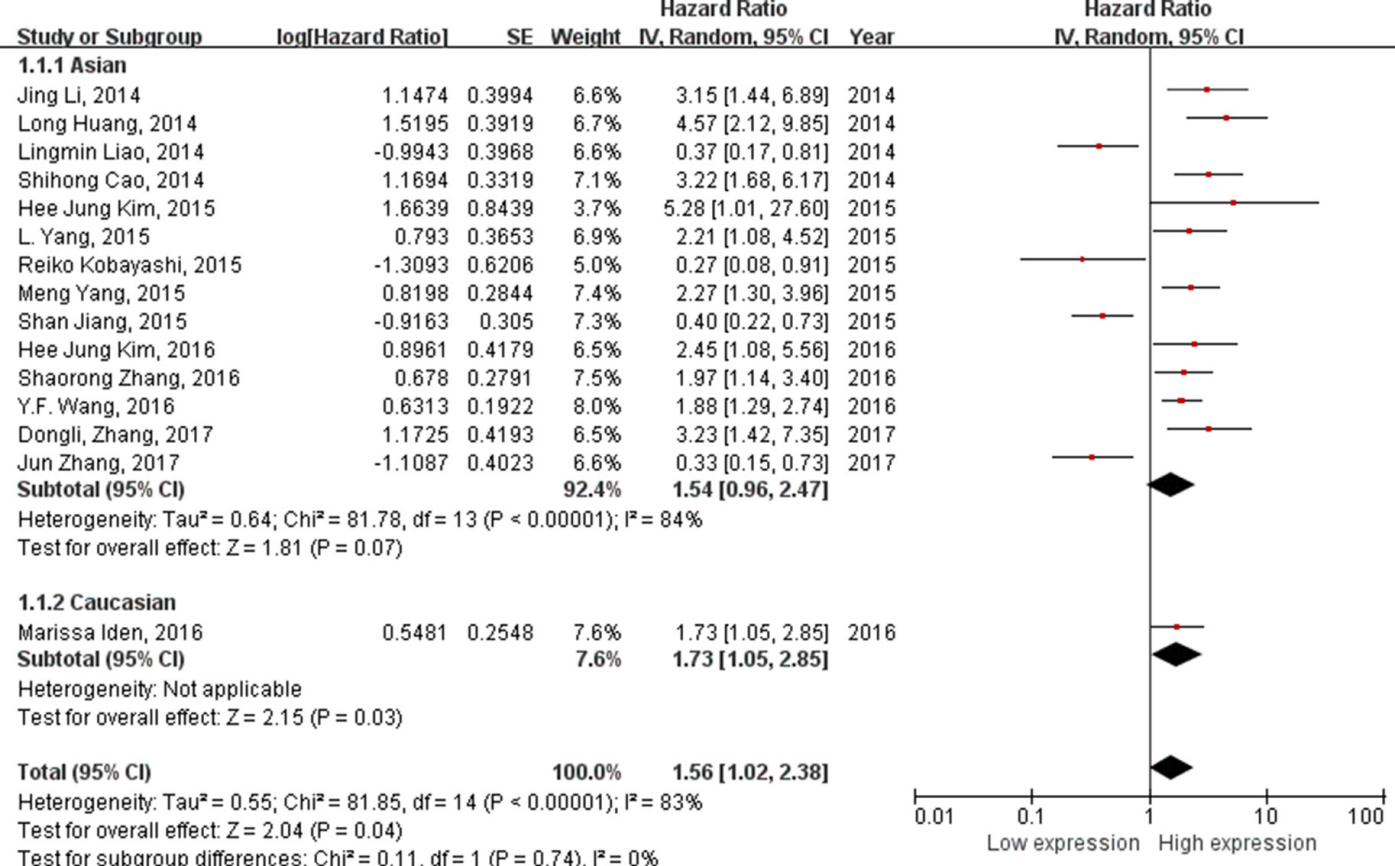
Forest plot of studies evaluating hazard ratios of lncRNAs expression and the overall survival in cervical The point estimate is bounded by a 95% confidence interval, and the perpendicular line represents no increased risk for the outcome.

A total of 15 studies were reported that the expression levels of lncRNAs were related to OS. The random-effect model was used to assess the HRs and 95%CI. As shown in Figure 2, the expression levels of lncRNAs were associated with the OS of cervical cancer patients (HR = 1.56, 95%CI = 1.02–2.38, *P* < 0.001, random-effect). From the forest plot, the high expressions of HOTAIR, PVT1, GAS5, MALAT1, CCHE1, HULC, HOXA11, ANRIL were associated with poor prognosis. Besides, XLOC_010588, XIST, LET, MEG3 were correlated to poor prognosis with the low expressions of lncRNAs in cervical cancer. With all the lncRNAs HOTAIR generate the highest HR of 5.28 [16]; by contrast, XIST exhibited the lowest HR of 0.27 [17]. Moreover, stratified analyses were performed using studies with the ethnicity. 14 articles reported the relationship between lncRNAs and Asian patents’ OS (HR = 1.54, 95%CI = 0.96–2.47, *P* < 0.01, random-effect), and 1article was about Caucasian (HR = 1.73, 95%CI = 1.05–2.85, *P* = 0.03, fixed-effect).

**Figure 3:**
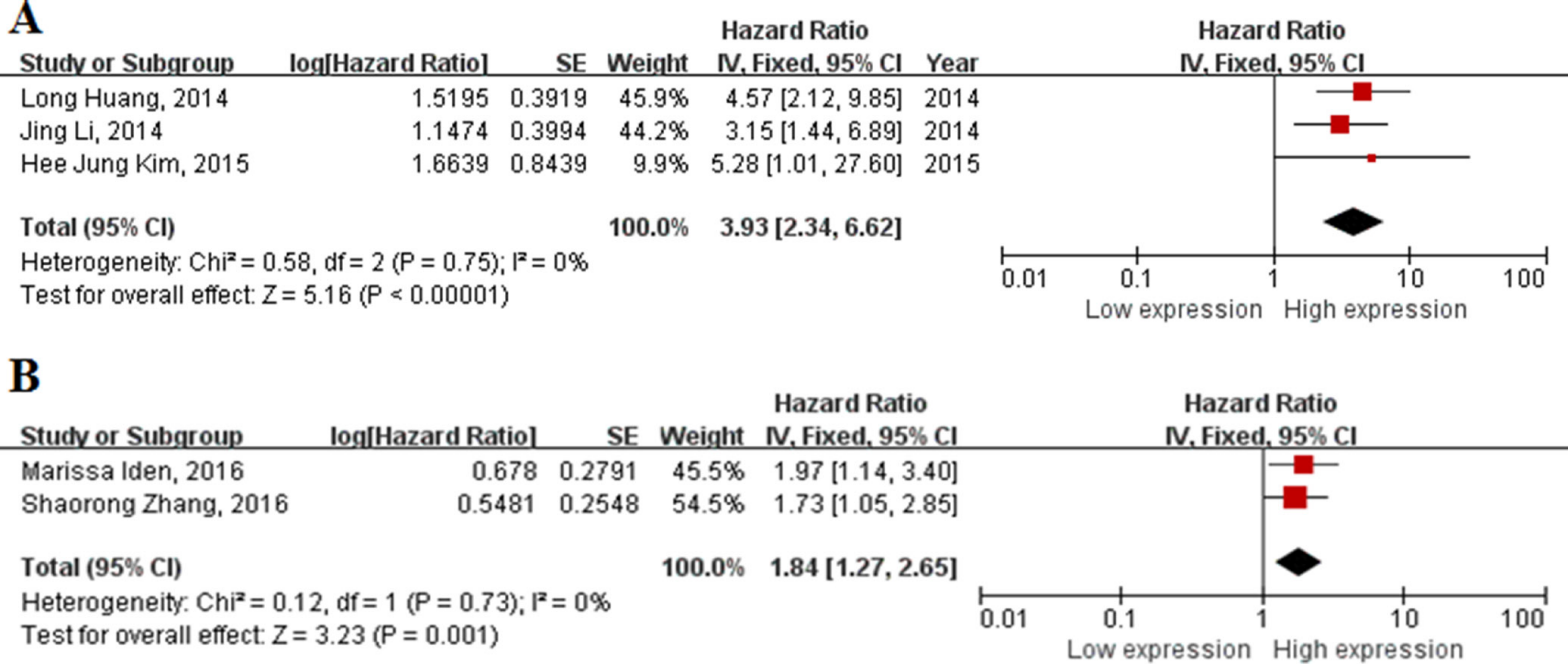
Forest plot of studies evaluating hazard ratios of up-regulated lncRNAs and the overall survival of cervical cancer (**A**) HOTAIR; (**B**) PVT1.

In the enrolling studies, HOTAIR and PVT1 were investigated in two or more articles, other lncRNAs were performed in single study. Then we carried out a meta-analysis on the relationship between the expressions of HOTAIR/PVT1 and the OS of patients with cervical cancer. We found that the high levels of HOTAIR were associate with a poor OS (HR = 3.93, 95% CI = 2.34–6.62, *P* < 0.001, fixed-effect) (Figure 3A). Meanwhile, a poor prognosis in cervical cancer was found in the increased of PVT1 (HR = 1.84, 95%CI = 1.27–2.65, *P* = 0.01, fixed-effect) (Figure 3B)

### Analysis between lncRNAs expression level and EFS

**Figure 4:**
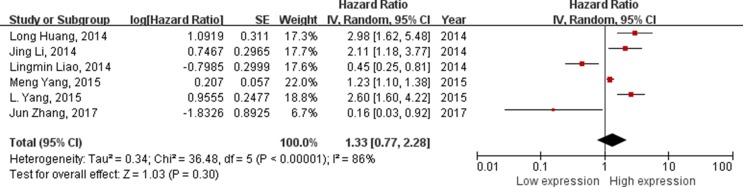
Forest plot f studies evaluating hazard ratios of lncRNAs expression and the event free survival in cervical cancer The point estimate is bounded by a 95% confdence interval, and the perpendicular line represents no increased risk for the outcome.

A total of 6 studies (912 patients) included in the EFS analysis revealed a protective role increased lncRNAs expression (HR = 1.33, 95%CI = 0.77–2.28, *P* < 0.01, random-effect) (Figure 4). From the forest plot, the increased expressions of HOTAIR, MALAT1 and CCHE1 correlated with a worse prognosis, the decreased expressions of XLOC_010588 and MEG3 were associated with a worse prognosis. With all the lncRNAs HOTAIR generate the highest HR of 2.98 [12]; by contrast, MEG3 exhibited the lowest HR of 0.16 [24].

### Publication bias and sensitivity analysis

**Figure 5:**
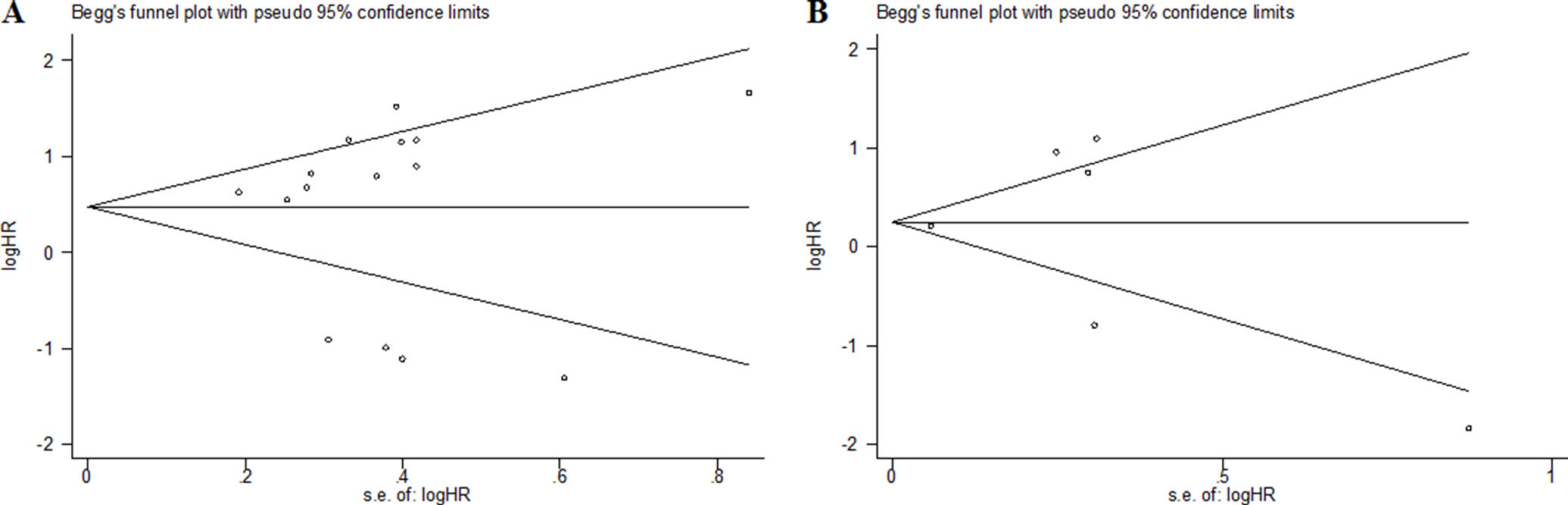
Begg’s test for publication bias (**A**) overall survival; (**B**) event free survival.

**Figure 6:**
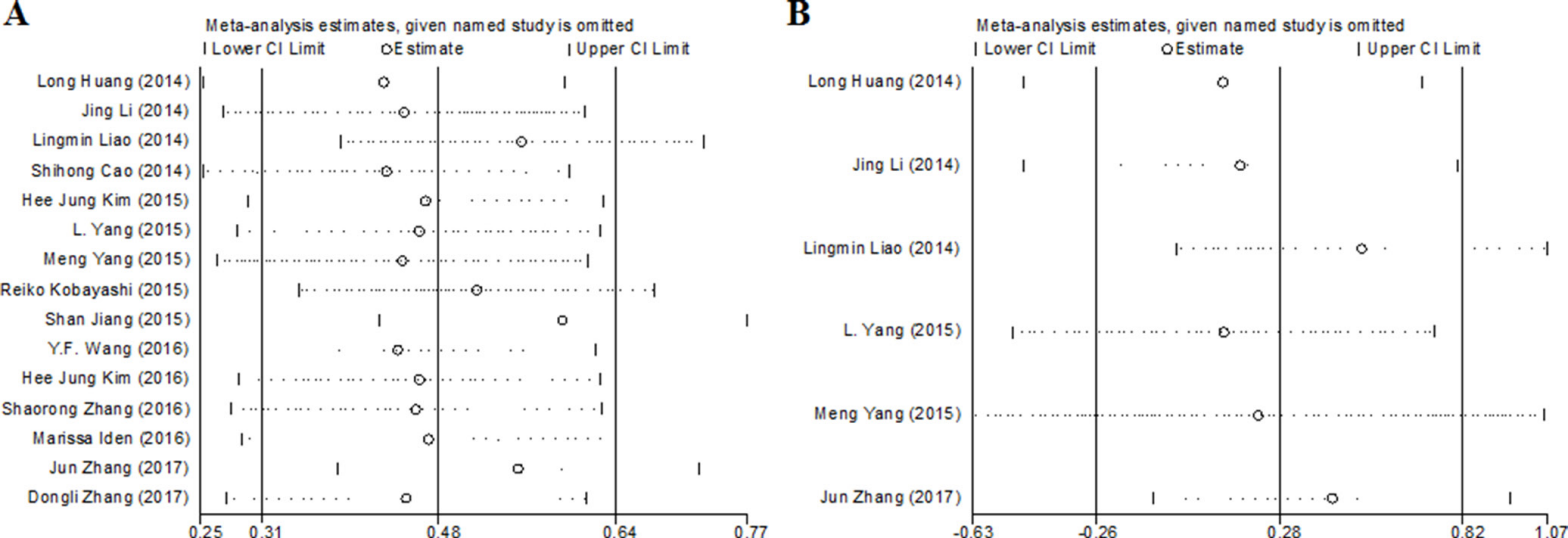
Sensitivity analyses of the studies (**A**) overall survival; (**B**) event free survival.

As shown in Figure 5, Begg’s test was used to perform the publication bias, respectively. In our meta-analysis, Begg’s test suggested there were no publication bias in all groups, owing to the values of *P* > 0.05. Meanwhile, we used Stata11.0 software to carry out sensitivity analysis to assess whether the individual studies affected the overall results. The results suggested that individual study had little influence on our eventual outcome (Figure 6), and proved that our analysis was relatively stable and credible.

## DISCUSSION

Cervical cancer is one of the leading causes of cancer related death in women worldwide [26]. Identification of effective disease biomarkers for prognosis is urgently required. In recent years, mounting epidemiological and molecular biological studies have demonstrated that lncRNAs dysregulation was involved in cancers [27–29]. Furthermore, abnormal expression of multiple lncRNAs was found to be related with the tumorigenesis and may have the potential to be prognostic biomarkers and even therapeutic targets of cervical cancer [15, 30]. Therefore, we perform this meta-analysis to evaluate the prognostic ability of lncRNAs in cervical cancer.

Up to present, two meta-analysis [30, 31] assessed the correlation between lncRNAs and cancer survival involved cervical cancer as one of the cancer sites. Both of these two studies focused on exploring a single lncRNA (H19 or HOTAIR) in relation to cervical cancer. With very different goal, our article mainly aimed to evaluate the prognosis ability of all lncRNAs in cervical cancer. Therefore, the current meta-analysis is the first to evaluate the relation between lncRNAs expression and prognosis of patients with cervical cancer comprehensively.

In current meta-analysis, we evaluated the prognostic value of lncRNAs in cervical cancer. Our results suggested that high expression of lncRNAs represented a risk factor for OS and cervical cancer. Furthermore, we found that the high expressions of 8 lncRNAs were associated with poor prognosis. Besides, 4 lncRNAs were correlated to poor prognosis with the low expressions of lncRNAs in cervical cancer. Moreover, stratified analyses were performed using studies with the ethnicity. 14 articles reported the relationship between lncRNAs and Asian patents’ OS (HR = 1.54,), and 1article was about Caucasian (HR = 1.73). Besides, EFS analysis also revealed a protective role decreased lncRNAs expression (HR = 1.33). Our results suggested that the expression of these lncRNAs may have a significantly prognosis value in cervical cancer. Due to the limitation of the study number, these conclusions need more clinical trials for verification.

In the enrolling studies, PVT1 and HOTAIR were investigated in two or more articles, and with the increased expression of the two lncRNAs, the prognosis rate was poor in cervical cancer. Our results revealed that PVT1 is increased in the cervical cancer patients, and the high expression of PVT1 was related with the poor prognostic outcome of cancer patients. Summary of Hazard ratios (HRs) of lncRNAs in cervical cancer patients showed that HOTAIR generate the highest HR of 5.28. HOTAIR was the first lncRNA found to be involved in tumor and has been proven to be raised in a variety of human cancers [32]. Meanwhile, HOTAIR is a tumorigenic factor and can be adopted as a diagnosis or predictive biomarker in various cancer types [33, 34], those findings in consist with our results. Notably, HOTAIR was the most investigated lncRNA in cervical cancer, therefore, articles explored HOTAIR as a new biomarker in the diagnosis and treatment of cervical cancer is possibly the most promising.

It should be stressed that there were limitation in our analysis. First, the number size of eligible articles is relatively small, which restrict our ability to evaluate the prognosis of lncRNAs in subgroup analyses. Second, the main ethnicities of the patients in our analysis were Asian, which also might affect the results. Third, due to positive results could be published more easily than negative results, this may lead to hidden publication bias.

In summary, the current meta-analysis was firstly to evaluate the correlation between lncRNAs and prognosis of patients with cervical cancer. Despite these limitations, there was a relationship between lncRNAs levels and OS and EFS in cervical cancer, which demonstrated the strong prognostic value of lncRNAs in cervical cancer. However, large-scale and comprehensive studies were needed to confirm our findings and thus promote the clinical utility of lncRNAs in cervical cancer prognosis evaluation.

## MATERIALS AND METHODS

### Search strategy

A comprehensive search was performed independently by two researchers (Xiangrong Cui and Xuan Jing) via PubMed, EMBASE and Web of Science for literatures published up to February 2017 to obtain relevant articles for the meta-analysis. The search strategy used both MeSH terminology and free text words to increase the sensitivity of the search. The keywords for the search in these databases included: “Long noncoding RNA”, “lncRNA”, “LincRNA”, “Long ncRNA”, “survival” and cervix (or cervical) cancer/neoplasm/tumour/carcinoma. Meanwhile, we screened the references of retrieved relevant articles to identify potentially eligible literatures.

### Inclusion and exclusion criteria

Literatures included in this meta-analysis had to meet the following inclusion criteria: studies about the association between lncRNAs expression in tissues or blood samples and prognosis of patients with cervical cancer; the survival outcomes were performed with OS or EFS including disease free survival DFS and RFS; patients were divided into high and low expression groups; sufficient published data were provided to calculate hazard ratios (HR) and 95% confidence interval (CI). Exclusion criteria were as follow: studies without usable or sufficient data; laboratory articles, reviews, letters, unpublished data and conference abstracts.

### Data extraction

Two investigators (Xiangrong Cui and Xuan Jing) extracted relevant data independently using predesigned and standardized form from the eligible studies. Extracted information included title, authors’ names, the nationality and ethnicity of study population, lncRNAs, case number, methodological information and cut-off value. Disagreements were resolved through group discussion.

### Statistical methods

HRs and 95% CIs were calculated to assess to the relation between lncRNAs and survival in cervical cancer, with a significance level of α = 0.05. An observed HR > 1 implied a worse survival for the group with elevated lncRNAs expression. Conversely, HR < 1 implied a worse survival for the group with decreased lncRNAs expression [35, 36]. We used Revman5.3 Software (Revman, the Cochrane Collaboration) to perform the meta-analysis and evaluate heterogeneity between studies by Cochrance *Q*-test and *P*-values. If heterogeneity was present (I^2^ ≥ 50% or *P* ≤ 0.05), random-effect model was used to calculate pooled HRs or ORs, otherwise, the fixed-effect model was utilized [37, 38]. Furthermore, we minimized the influence of heterogeneity through classifying the included studies into subgroups accorded to similar features. The Stata 11.0 Software (Stata, College Station) was performed to evaluate the sensitivity and publication bias of the studies. Publication bias was estimated using Begg’s test with a funnel plot, *P* < 0.05 was considered statistically significant.
